# Isolated Dorsal Trapezoid Dislocation at the Scaphotrapeziotrapezoidal Joint: A Case Report and Literature Review

**DOI:** 10.7759/cureus.111065

**Published:** 2026-06-17

**Authors:** Laxman Choudhary, Syed Mohammed Adnan, Amir Suhail, Furqan Meer, Nitesh Gahlot

**Affiliations:** 1 Orthopaedic Surgery, All India Institute of Medical Sciences, Jodhpur, Jodhpur, IND; 2 Orthopaedic Surgery, Sawai Man Singh Hospital (SMS), Jaipur, IND; 3 Orthopaedic Surgery, Government Medical College, Pali, IND; 4 Orthopaedics and Trauma, All India Institute of Medical Sciences, Jodhpur, Jodhpur, IND; 5 Orthopaedics, All India Institute of Medical Sciences, Jodhpur, Jodhpur, IND

**Keywords:** carpal alignment, carpal injury, multiplanar reconstruction, scaphotrapeziotrapezoidal (stt) joint, trapezoid dislocation

## Abstract

Isolated dorsal dislocation of the trapezoid at the scaphotrapeziotrapezoidal (STT) joint represents an exceptionally rare entity among carpal injuries. This report documents the case of an adult male who sustained an injury in a high-energy road traffic accident and presented with wrist pain, swelling, and deformity. Radiographs and computed tomography confirmed a dorsal dislocation of the trapezoid at the STT joint without associated perilunate disruption or carpal fractures. The injury was successfully managed with closed reduction and splint immobilization. At the short-term follow-up, the patient demonstrated anatomic alignment, full recovery of wrist function, and completely pain-free movement. This case highlights the importance of maintaining a high index of suspicion and utilizing advanced imaging to ensure early recognition and optimal treatment of this rare carpal injury.

## Introduction

Carpal dislocations are relatively uncommon orthopedic injuries typically resulting from high-energy trauma [1]. Within this subset, isolated dislocation of the trapezoid bone-historically referred to as the lesser multangular-is exceptionally rare, accounting for less than 1% of all carpal injuries [2,3]. This remarkable stability is derived from the trapezoid's position as a "keystone" in the distal carpal row, where it is tightly wedged between the scaphoid, capitate, trapezium, and the base of the second metacarpal. It is further secured by a robust network of dorsal and palmar intercarpal ligaments that resist displacement [2,4].

Considerable controversy persists regarding optimal management, specifically concerning the clinical choice between immediate closed reduction and primary surgical fixation. Proponents of acute closed reduction and immobilization argue that it effectively preserves joint mechanics and minimizes surgical morbidity in stable, acute presentations. Conversely, advocate groups for primary open reduction and internal fixation, often utilizing Kirschner wires or headless compression screws, emphasize that severe capsuloligamentous disruptions inherently compromise long-term carpal stability, predisposing the patient to late subluxation, chronic pain, and secondary osteoarthritis if not rigidly fixed.

Recent literature emphasizes that the primary mechanism for this injury is a significant axial load transmitted indirectly through the second metacarpal, often involving forced wrist flexion or extension [5]. Although dorsal dislocations are more prevalent, rare cases of palmar displacement have been documented, often following complex shear forces [6-8]. Due to the rarity of the injury and the subtlety of plain radiographic findings, such as minor disruptions in Gilula’s carpal arcs, these dislocations are frequently missed or misdiagnosed during initial evaluations [9]. Advanced imaging, specifically computed tomography, is therefore essential to confirm the diagnosis and assess for concomitant carpal fractures [10]. Prompt recognition and anatomical reduction are critical, as delayed treatment is associated with debilitating long-term sequelae, including chronic wrist pain, diminished grip strength, and progressive secondary osteoarthritis [7,11].

## Case presentation

Case

A previously healthy 30-year-old male farmer was admitted to the emergency department following a high-energy motor vehicle collision involving a motorcycle and a four-wheeled vehicle. The patient sustained a direct, high-impact blow to the dorsum of his dominant hand. He presented with an immediate onset of acute pain, significant swelling, and a visible deformity, resulting in a total inability to perform functional wrist movements. There was no significant past medical history.

Physical examination revealed marked soft tissue edema and focal tenderness localized to the dorso-radial aspect of the carpus. At the base of the second metacarpal, a distinct, firm, and non-mobile bony prominence was felt. This was a "step-off" deformity. This finding is highly suggestive of dorsal displacement of the trapezoid, historically referred to as the lesser multangular bone. Both active and passive ranges of motion were severely restricted in all planes due to mechanical blockage at the scaphotrapeziotrapezoidal joint and associated pain.

The overlying skin remained intact, with no signs of open injury or impending compartment syndrome. A comprehensive neurovascular assessment confirmed that the median, ulnar, and radial nerves were intact, with preserved distal sensation and motor strength. Distal perfusion was stable, with brisk capillary refill and palpable radial and ulnar pulses. The clinical presentation was consistent with an isolated carpal dislocation, a rare occurrence given the trapezoid's stable position within the distal carpal row

Investigations

The first X-ray test of the wrist included standard anteroposterior, lateral, and oblique views (Figure 1). The AP radiograph demonstrated a disruption of Gilula’s carpal arcs, with subtle overlapping of the trapezoid on the adjacent scaphoid and capitate, and a loss of the normal joint space at the base of the second metacarpal. The lateral view confirmed a dorsal displacement of the trapezoid relative to the distal carpal row, a finding characteristic of this rare injury.

**Figure 1:**
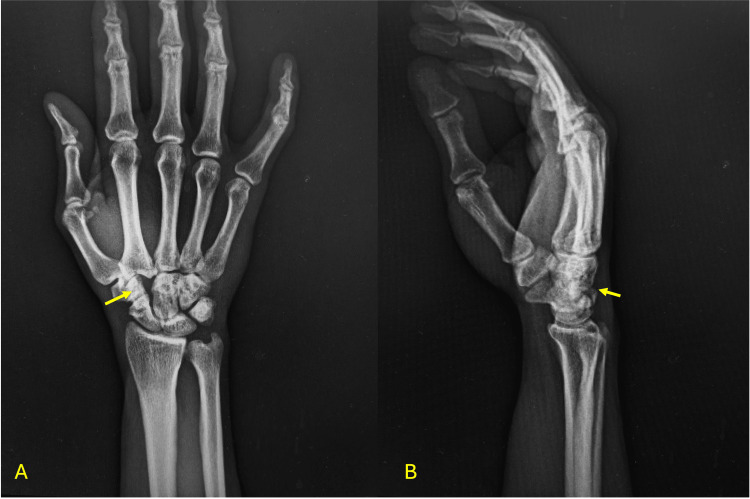
Initial plain radiographs of the injured wrist The posteroanterior and lateral views (A, B) display a distinct disruption of Gilula's carpal arcs and an asymmetrical step-off in the distal carpal row. The yellow arrow points to the dorsal displacement of the trapezoid bone as it sits completely dissociated from its anatomical position at the scaphotrapeziotrapezoidal (STT) joint. The second metacarpal base appears misaligned, with a distinct step-off noted at the central carpal region. The trapezoid is visualized as being elevated dorsally, interrupting the continuity of the distal carpal row. This image does not show any obvious fractures of adjacent carpal bones, and the rest of the carpal bones appear to maintain gross anatomical relationships without perilunate dislocation. The dorsal prominence corresponds clinically to the palpable bump described on examination prior to reduction.

Because the anatomy of the wrist is so complicated and there could be hidden fractures, a non-contrast computed tomography scan (Figure 2) of the wrist was done. The CT scan definitively confirmed an isolated dorsal dislocation of the trapezoid at the scaphotrapeziotrapezoidal joint. Three-dimensional (3D) reconstructions were utilized to precisely map the spatial orientation of the displaced trapezoid and to rule out concomitant carpal fractures or perilunate instability, which are common in high-energy wrist traumas. No associated fractures of the scaphoid, capitate, or second metacarpal base were identified, confirming the diagnosis as an isolated trapezoidal dislocation.

**Figure 2:**
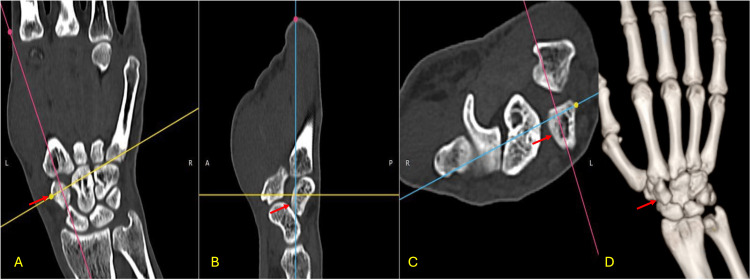
Non-contrast computed tomography (CT) scan of the wrist for definitive evaluation A. Coronal View: This section cuts through the carpus and clearly displays the dorsal displacement of the trapezoid at the STT joint (red arrow). The trapezoid is located superolaterally compared to its normal articulation with the scaphoid and the base of the second metacarpal. Surrounding carpal bones, including the scaphoid, capitate, and trapezium, retain their structure, ruling out associated fractures. B. Sagittal View: This perspective accentuates the displacement, with the trapezoid seen projecting dorsally (red arrow), sitting above the carpal plane. The joint space at the STT interface is widened. The trapezoid is isolated from the remainder of the carpus, confirming dislocation rather than subluxation. C. Axial View: The cross-sectional image shows the trapezoid shifted dorsally and distinct from the adjacent carpal bones (red arrow), validating the abnormal positioning seen in two other planes. There is an absence of intra-articular fragments or complex carpal disruptions, and the overall marrow and cortical outlines of the trapezoid and neighboring bones are intact. D. 3D CT shows the trapezoid to be dorso-laterally displaced without any joint congruity (red arrow) with the scaphoid and base of the second metacarpal.

Magnetic resonance imaging was not considered essential in the acute phase due to increased edema but is still a potential option for evaluating ligamentous integrity in chronic or enduring symptomatic instances.

Treatment

After the wrist was stabilized, it was put in a removable splint and raised to reduce swelling. Analgesic and anti-inflammatory medications were administered for pain control, and the limb was closely monitored for evolving neurovascular compromise; no such complications developed during the observation period.

Given the absence of open injuries, associated fractures, or gross instability, closed reduction under conscious sedation was selected as the primary intervention. Current evidence supports this approach, as the preferred initial strategy for acute, isolated trapezoid dislocations without significant delay in presentation [12]. The reduction technique involved applying longitudinal traction along the axis of the wrist and fingers with countertraction at the forearm. Simultaneously, directed dorsal pressure was applied to the displaced trapezoid while volar counterpressure was delivered at the base of the second metacarpal. A distinct palpable "click" was appreciated, signifying successful reduction, followed by immediate restoration of normal wrist contours.

Post-reduction radiographs (Figure 3) and computed tomography confirmed congruent reduction of the trapezoid and restoration of normal carpal alignment. The limb was subsequently immobilized in a below-elbow slab with the wrist positioned in slight extension for four weeks. This duration of immobilization is consistent with reported protocols for carpal dislocations, which typically recommend three to six weeks of cast or splint immobilization to allow ligamentous healing while minimizing stiffness [12,13].

**Figure 3:**
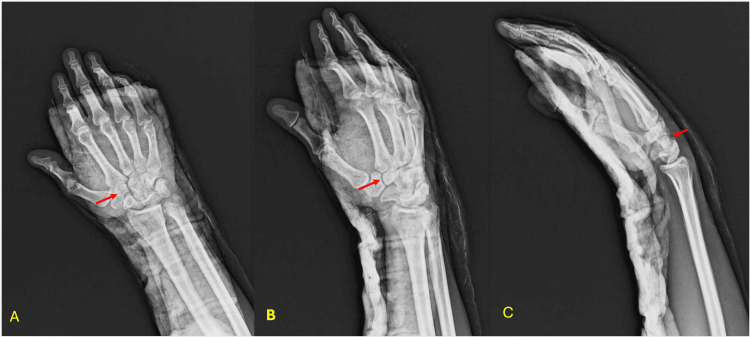
Immediate post-reduction plain radiographs of the wrist The posteroanterior, oblique, and lateral (A, B, and C) X-rays of the wrist show successful anatomical realignment of the trapezoid within the STT joint. The base of the second metacarpal is now appropriately articulated with the trapezoid (red arrow), and the trapezoid has returned to its normal parallelogram configuration within the distal carpal row (red arrow). The red arrow demonstrates the successful restoration of Gilula's carpal arcs and the correction of the previous step-off deformity at the base of the second metacarpal.

Outcome and follow-up

At two weeks, there was no recurrence of deformity or swelling. The patient reported mild to moderate post-traumatic stiffness but was pain-free at rest and on gentle movement. No neurovascular deficits were detected. Repeat radiographs confirmed the maintained reduction. After cast removal at four weeks, supervised graduated wrist range-of-motion and grip-strengthening exercises were initiated. At three months post-injury, the patient demonstrated nearly full, pain-free range of motion at the wrist and hand. There was no recurrence of instability, subluxation, or neurological symptoms. The patient returned to his pre-injury functional status without the need for any further intervention.

At the extended follow-up evaluation of 12 months post-injury, the patient’s clinical status remained excellent, demonstrating long-term carpal stability without any secondary deterioration. Objective functional outcome measures were obtained to rigorously quantify his recovery. Grip strength testing performed with a hand dynamometer revealed that the patient achieved 95% of the strength of his uninjured, contralateral dominant hand (48 kg vs. 50 kg). Standardized patient-reported outcome scores further corroborated this functional recovery, with the patient scoring a 2.3 on the Disabilities of the Arm, Shoulder and Hand (DASH) questionnaire and a 96 out of 100 on the Patient-Rated Wrist Evaluation (PRWE), both indicating minimal residual disability and excellent long-term adaptation [14,15]. The patient has fully returned to his heavy agricultural duties as a farmer without experiencing any pain, mechanical click, or subjective weakness.

## Discussion

Epidemiology and anatomy

Isolated dislocation of the trapezoid is an exceptionally rare clinical event, representing less than 1% of all carpal injuries. The trapezoid, historically referred to as the lesser multangular bone, is the least frequently injured carpal bone, accounting for approximately 4% of carpal fractures [5]. This is rare because it is the "keystone" of the distal carpal row. It is securely wedged in a rigid osseous pocket, articulating with the scaphoid proximally, the trapezium laterally, the capitate medially, and the base of the second metacarpal distally [2]. Its stability is further reinforced by a dense network of strong dorsal and palmar intercarpal ligaments, which typically require high-energy forces to overcome (Figure 4) [2].

**Figure 4:**
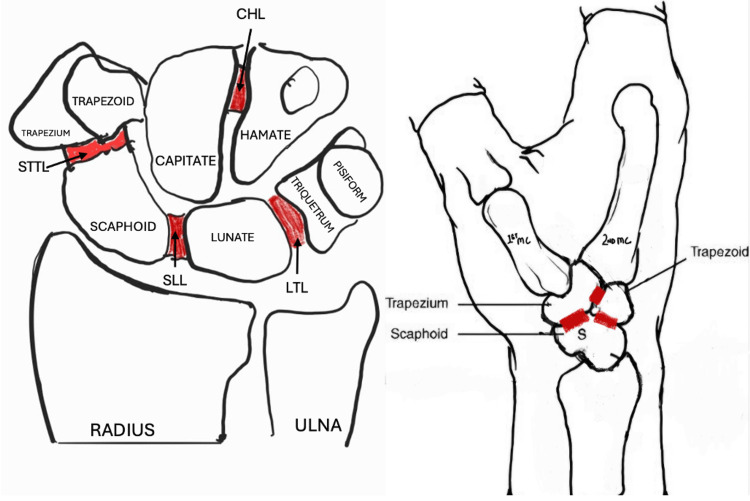
Anatomical reference diagram demonstrating carpal ligamentous architecture and the complex biomechanics of the scaphotrapeziotrapezoidal (STT) joint The left panel provides an anterior (volar) coronal view of the carpus, illustrating the central, nested "keystone" position of the trapezoid bone within the distal carpal row, securely bound by primary intrinsic stabilizing ligaments (highlighted in red). The right panel illustrates the focal ligamentous constraints of the STT joint complex and its relationship to the index (second metacarpal) and thumb (first metacarpal) rays, demonstrating how indirect axial loads are transmitted retrogradely through the metacarpal base to isolate or disrupt these specific joint interfaces. Abbreviations: STTL: Scaphotrapeziotrapezoidal ligament; SLL: Scapholunate ligament; LTL: Lunotriquetral ligament; CHL: Capitohamate ligament; S: Scaphoid; 1st MC: First metacarpal; 2nd MC: Second metacarpal.

Pathophysiology and injury mechanism

The biomechanical forces required to displace the trapezoid are significant. The primary mechanism involves a high-magnitude axial load transmitted through the second metacarpal, often during high-impact trauma such as motorcycle collisions [5]. This force vector typically results in a dorsal displacement, as seen in this case, although palmar dislocations have been documented, frequently involving complex shearing forces and associated coronal fractures [7,10]. Unlike more common perilunate dislocations that follow a predictable sequence of ligamentous failure, isolated trapezoid dislocation suggests a focal disruption of the scaphotrapeziotrapezoidal and trapezio-capitate ligamentous complexes [1,3].

Diagnostic challenges and imaging

Clinical diagnosis is notoriously difficult due to the bone's deep position and the rapid onset of diffuse swelling that masks bony landmarks. While a palpable "step-off" at the base of the second metacarpal is a suggestive clinical sign, it is often missed in the acute setting [2]. Standard radiographs are frequently inconclusive because the trapezoid's small size and position lead to overlapping carpal shadows on AP and lateral views [5]. Radiographic diagnosis relies on identifying a disruption of Gilula’s carpal arcs or subtle joint-space widening [9]. Computed tomography, particularly with 3D reconstruction, is the gold standard for management; it not only confirms the direction of dislocation but also identifies occult fractures of the capitate or scaphoid, which are common in high-energy wrist trauma [5,10].

Management and prognostic factors

Management strategies for this uncommon injury remain unstandardized owing to the absence of extensive prospective studies. While acute dorsal dislocations can often be managed with closed reduction, evidenced by a palpable "click" during longitudinal traction and dorsal-to-palmar pressure, the stability of the reduction must be scrutinized [2,12]. The reduction is stable; a period of four to six weeks of cast immobilization is typically recommended to allow ligamentous healing. However, some authors advocate for percutaneous K-wire fixation to provide superior stability and prevent late displacement [16].

Open reduction is indicated when closed attempts fail, often due to interposition of the dorsal capsule or ligamentous fragments [12,16]. Failure to achieve an anatomical, concentric reduction significantly increases the risk of chronic pain, reduced grip strength, and rapid progression of secondary osteoarthritis at the scaphotrapeziotrapezoidal or carpometacarpal joints [7,8]. Long-term follow-up is essential to monitor for avascular necrosis, although the trapezoid's multiple articular surfaces and ligamentous attachments usually provide a redundant blood supply compared to the scaphoid [5].

Choosing non-operative closed reduction for an acute carpal dislocation requires a critical evaluation of joint stability, a decision that remains a point of contention when contrasted with previously published operative workflows. A review of the literature reveals that authors like Rhoades and Reckling and Calfee et al. bypassed closed pathways altogether, proceeding directly to open reduction due to mechanical blocks from interposing capsuloligamentous fragments or associated coronal shearing fractures. Furthermore, Maxwell and Morris advocated for percutaneous Kirschner-wire transfixion even after a successful closed reduction, arguing that unpinned ligamentous complexes inherently invite progressive carpal collapse or subtle instability under physiological loads.

Our clinical reasoning deviated from these operative strategies because our patient presented acutely, possessed completely intact native margins without coronal shearing fragments on multiplanar CT, and achieved an extraordinarily stable, concentric reduction immediately upon manual manipulation. While primary internal transfixion or open capsular repair provides rigid mechanical immobilization, it introduces risks of pin-tract infection, tendon irritation, and prolonged joint arthrofibrosis. By choosing immediate non-invasive closed reduction and temporary below-elbow cast immobilization, we successfully avoided these surgical morbidities. Our long-term objective metrics confirm that when an isolated dorsal trapezoid dislocation is strictly concentric and structurally stable post-reduction, the native intercarpal constraints possess sufficient healing potential to restore full grip strength and prevent proximal carpal migration without invasive hardware.

Clinical implications and future directions

This case underscores the necessity of a high index of clinical suspicion in high-energy wrist injuries with persistent localized tenderness. Future research, perhaps utilizing finite element analysis and biomechanical modeling, could better elucidate the specific trauma vectors leading to isolated carpal dislocations. From a patient-centered viewpoint, prompt diagnosis and anatomical restoration are essential for reinstating patients to their baseline occupational and athletic pursuits, thereby alleviating the socioeconomic ramifications of chronic wrist instability and early-onset degenerative disease.

Literature review

A comprehensive review of the documented cases in the literature emphasizes that isolated trapezoid dislocations are rare, representing a small fraction of all carpal injuries. The diagnostic and therapeutic data compiled from historically relevant and recent publications are summarized below in Table 1.

**Table 1:** Clinical characteristics and treatment outcomes of isolated trapezoid dislocations reported in the literature Data compiled from reliably documented cases of isolated trapezoid injuries. The summary highlights the high correlation between high-energy trauma vectors and dorsal patterns of displacement, alongside the corresponding success rates of early anatomical reduction. ROM = range of motion; XR: X-ray; CT: computed tomography

Author	Dislocation Direction	Primary Mechanism	Diagnostic Imaging	Associated Injuries / Fractures	Management Strategy	Immobilization Duration	Follow-up Duration & Functional Outcome
Stein [2]	Dorsal	Motor vehicle collision	XR	None (Isolated)	Closed reduction under conscious sedation	4-week cast	6 months; excellent, pain-free restoration of full wrist motion
Maxwell & Morris [3]	Dorsal	Motorcycle accident; retrograde axial load	XR & CT	None (Isolated)	Closed reduction followed by percutaneous K-wire transfixion	4-week splint	8 months; stable carpal alignment, complete return to baseline function
Nammour et al. [5]	Dorsal	Indirect axial force through the second ray	XR & CT	Non-displaced chip fracture of capitate	CT-guided surgical planning and percutaneous screw fixation	4-week splint	12 months; complete resolution of symptoms, highlighting critical reliance on CT
Calfee et al. [10]	Palmar	High-energy blast; forced wrist hyperextension	XR & CT	Concomitant coronal shearing fracture of trapezoid body	Open reduction and internal fixation (ORIF) with headless screws	6-week splint	18 months; pain-free functional ROM, no signs of post-traumatic arthritis
Taylor & Shakespeare [17]	Dorsal	High-energy axial forearm force	XR	Ipsilateral Galeazzi fracture-dislocation	Closed reduction of carpus and forearm stabilization	5-week cast	6 months; successful carpal reduction maintained, stable joint arcs
Meyn & Roth [18]	Dorsal	Direct crush injury	XR	None (Isolated)	Closed reduction and manual manipulation	6-week cast	12 months; excellent functional recovery, normal grip strength
Rhoades & Reckling [7]	Palmar	High-impact direct blow to the dorsal hand	XR	Soft tissue tearing; palmar capsular avulsion	Open reduction required due to severe ligamentous interposition	4-week cast	24 months; good functional recovery, full restoration of grip strength
Koenig & West [6]	Palmar	Fall onto an outstretched hand	XR & CT	None (Isolated)	Open reduction via volar approach and K-wire transfixion	6-week splint	14 months; stable carpus, mild residual terminal stiffness on heavy lifting

In summary, isolated carpal bone dislocation should always be considered in patients presenting with high-energy wrist trauma and unexplained carpal malalignment on plain films. A meticulous assessment of Gilula’s arcs alongside prompt utilization of CT imaging remains crucial for ensuring an accurate diagnosis. When managed early, acute, reducible, and isolated dorsal trapezoid dislocations respond highly effectively to closed reduction and cast immobilization, yielding excellent functional recovery with minimal sequelae. Ultimately, educating frontline healthcare providers and broadening access to advanced imaging can significantly mitigate the risk of missed or delayed diagnoses in hand trauma.

## Conclusions

In conclusion, isolated trapezoid dislocation, despite its rarity, requires prompt and accurate diagnosis, primarily through advanced imaging, such as CT, to ensure optimal patient outcomes and prevent long-term functional deficits. The successful management of the reported case reinforces the efficacy of closed reduction and short-term immobilization for stable dislocations, emphasizing careful post-reduction stability assessment.
